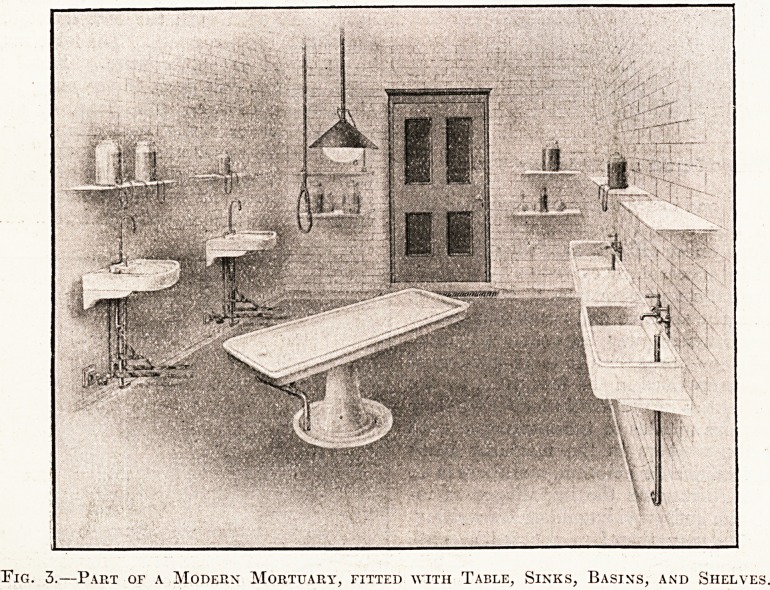# The Mortuary Unit

**Published:** 1913-05-17

**Authors:** 


### THE MAKING OF A MODERN HOSPITAL.

### The Mortuary Unit.

### THE FITTINGS AND EQUIPMENT OF THE POST-MORTEM ROOM.—V.

IN a well-provided modern hospital considerable attention is paid to the equipment of the mortuary unit, for it is recognised very clearly that unless

FIG. 1.—THE "IMPERIAL" MORTUARY TABLE.

modern apparatus and instruments are furnished the work done in this special department must of necessity be done under difficulties. These difficulties can be and are successfully overcome by workers who are enthusiastic and painstaking, but only at the expense of inconvenience and waste of valuable time and energy which may be conserved more usefully for other purposes than to make good what should have been provided in the first place by the responsible authorities of the institution. We have referred in previous articles to the imperative need for providing the workers in the mortuary block with abundant facilities for ablution, and there is no further need to insist upon the importance of installing good baths and providing adequate waiting-room space. We may therefore proceed at once to discuss the equipment of the mortuary proper, and especially of its most important constituent, the post-mortem room or section room in which the work of actual autopsy is carried on.

THE ESSENTIAL FITTINGS.

In the previous article we have outlined the requirements of this working room so far as lighting, ventilation, and heating are concerned, and we have referred to additional points, such as the character and nature of the flooring, the impermeability of the wall surfaces, and the desirability of having strict privacy, such seclusion being secured by proper isolation of the public from the private part of the unit. With regard to the essential fittings, these should be simple but efficient and durable; a little extra expense incurred upon modern appliances of first-class workmanship is in the end good economy, since such appliances last longer, are more satisfactory, and really simpler, in that they do not get out of order or need frequent repairs, than cheaper and inferior quality articles. Obviously the most important item is the mortuary table on which the body rests for autopsy purposes. There are different styles of table, but those most generally used have certain characteristics which are common to all.

THE TABLE.

They are made of some hard substance which is impermeable to fluids, acids, or corrosives, and which can be easily cleaned by warm liquids or antiseptic solutions of moderate strength. This is the first consideration. A wooden table, for example, is quite out of place in a modern mortuary; it absorbs moisture and odour, is cleaned with difficulty, and even with the best dovetailing is quite impossible to keep sweet. The table must therefore be of metal or earthenware. Metal tables were used, and are still used, in the older hospitals, the slabs being of iron, zinc, lead, or alloy. While such tables are easily kept clean, they soon lose their pristine appearance and look dirty and worn; they are rarely impervious to the action of acids and corrosives, and apt to get oxidised after a time, while if they are equipped with labour-saving devices

FIG. 2.—MORTUARY WASH-BASIN, WITH DOUBLE TREADLE ACTION.

for lowering or raising the slabs, they wear badly and are difficult to keep clean in the interstices and joints. For these reasons the earthenware table has of recent years been recognised as the ideal. It is strong, absolutely impermeable so far as surface absorption is concerned, easy to clean and keep in good order, and usually wears remarkably well, so that a table after several years' continuous use still looks fresh and new. Against it is the comparatively high price, but in view of the fact that it is practically indestructible with ordinary care, it is much better for an institution to invest in a table of this kind than to acquire the old-fashioned metal-slab table which is still seen in many mortuaries.

### Types and Details.

Various types of the earthenware table are listed in dealers' catalogues, and the choice is a wide one. Without wishing to recommend one type above another, we may here draw attention to the excellent tables and mortuary fittings manufactured by the Leeds Fireclay Company, Limited, of Wortley, Leeds, who supply the "Cliff" fittings patented by Joseph Cliff and Sons, of Leeds. Tables of this type have been fixed recently in the post-mortem rooms at the London Hospital and have given every satisfaction. The best pattern undoubtedly is the "Imperial," which is made of glazed white porcelain on a glazed pedestal, with cast-iron internal ball bearings for easy movement, set screw for adjustment of the slab into any position desired, and porcelain base and channel, combined with gun-metal locking gate. The prices charged for this table, considering the excellence of the workmanship and the quality of the whole, are extremely moderate. Fig. 1 shows such a table, from which it can be observed that the shape is simple and that the whole fulfils every requirement, so far as appearance is concerned, of an ideal mortuary table. Mortuary slabs of this "Imperial" type are also supplied by the same firm and are useful adjuncts, though they are fixed. We ourselves prefer, for examining purposes, a plain glass slab, diagonally or horizontally divided into two differently coloured parts, the one of plain greenish plate-glass and the other black. Such an arrangement considerably facilitates the examination of organs and sections under different conditions of refracted light, and is a very useful and by no means expensive adjunct.

### The Question of Number.

Proper arrangements must be made to allow for washing specimens while on the table or slab. A rubber hose is perhaps the best; it can be carried to any part and does away with fittings to the table or slab, since the tap can be fixed to the wall; in that case a counter nozzle tap must be provided so as to regulate the flow of the water at the exit in case of necessity. The number of tables and slabs provided will, of course, vary with the amount of work to be done in the department; if there are from three to six autopsies per day—the average number in a hospital of, say, four hundred beds—one table and two slabs are quite sufficient, unless demonstrations are given. In the latter case an additional table must be provided. If there is good chilling apparatus and the post-mortem workers know their business thoroughly, one table is quite sufficient for ordinary purposes. There is no necessity whatever for exposing the bodies on separate tables, each awaiting its turn. At the most a couple of slabs should be provided on which the body already examined is placed for the post-mortem room attendant to sew up and prepare, while the demonstrator proceeds with his work on the other corpse. Nothing, we may add, can

Fig. 3.—Part of a Modern Mortuary, fitted with Table, Sinks, Basins, and Shelves.

be more undignified to all concerned than to hurry unnecessarily over a post-mortem examination; if the arrangements in the mortuary are good and up to date there will be no desire on the part of the demonstrator to hasten his work. The scurry and bustle to be seen in some hospital post-mortem rooms are due to the fact that both students and demonstrators look upon the autopsies as very unpleasant routine work which must be got through in the minimum amount of time. With a conscientious demonstrator this does not matter, but there is reason to believe that post-mortem room statistics are vitiated very often by serious omissions in the examination due to the want of method and the hurry with which the autopsies are conducted. It is not an exaggeration to say that every autopsy, to be of real scientific value, should be done with the same care and precision as a so-called medico-legal post-mortem.

### Sinks and Washing-basins.

In the post-mortem room itself sinks and washing-basins should be provided with hot and cold water taps. Each washing-basin should have an arrangement whereby the temperature of the water issuing from the "hot" tap may be regulated. Fig. 2 shows a useful lavatory basin made by Oates and Green and supplied by the Leeds Fireclay Company, Limited. It is rectangular in shape, and made of white glazed fireclay, with the corners rounded to a small radius; it is supported on vitreous enamelled cast-iron brackets and fitted with gun-metal curved supply pipe with hot and cold treadle action valves and combined standing waste and overflow to floor pipe. Fig. 3 shows the interior of a model post-mortem room fitted with table, sinks, basins, and shelves. The examining slabs are not shown, and the position of the hose pipe fixed straight above the table itself is bad, but otherwise the arrangement is on the whole good. Care must be taken that there is adequate overhead light above the examining slabs, which must be placed conveniently close to the table.

The instruments necessary for post-mortem work are simple and not very numerous. The points most often overlooked are the sharpness of the knives and the make of the handles. Wooden-handled instruments are an abomination here as in the operating theatre on account of the difficulty of cleaning them; the only wooden instrument which it is justifiable to use is the mallet, and even here—although post-mortem room attendants, we know, are unlikely to agree with us—we prefer to use a moderately sized steel mallet of the type used in bone work in the theatre. Wire saws of the Gigli type should be provided, since they considerably facilitate certain explorations of the skull, the resultant cuts being small and easily concealed, whereas the cuts made by the ordinary saws are more gross.

### The Head-rest.

An important instrument, often overlooked in equipping a post-mortem room, is a head-rest; this is used in sawing open the skull to get at the brain, and is very useful if the work is to be done expeditiously and nimbly. Most attendants still practise the old-fashioned circular method of removing the skull cap; a triangular section is much more useful, since it effectually conceals afterwards the fact that the skull has been opened. These are small points, but they are worth insisting upon, since it must be the endeavour of those who do post-mortem work to yield the bodies to the relatives and friends in as normal a state as possible, and it is therefore highly desirable that all unnecessary mutilation should be avoided. Many of the manipulations now roughly done by the attendants should be carried out by the demonstrator himself; some of them are in the nature of delicate surgical operations and should be done with the same care and attention to detail that are demanded in a surgeon who carries out an operation in the theatre. The actual sewing up of the body after the viscera have been replaced is done by the attendants on a special slab; the trolley must not be used for the body while this operation is done, since it is only intended for the conveyance of clean bodies to and from the room.

### Photographing Specimens.

It is well to provide in every post-mortem room, at the far end of the room and in a position where the direct light is good, a special slab that is fixed to the wall and that can be inclined at various angles; this serves admirably for the photographing of certain specimens that cannot be taken away. Of course, ordinary photographic work must be carried out in a special room which adjoins the demonstrators' work room or library; special provision for such a photographic atelier is made in Vandervelde and Depage's plan, which has already been given. In the same room sketches and drawings are made. This work cannot conveniently be carried out in the post-mortem room, but immediate photographs or sketches may be made with the help of the special slab.

## Figures and Tables

**Fig. 1. f1:**
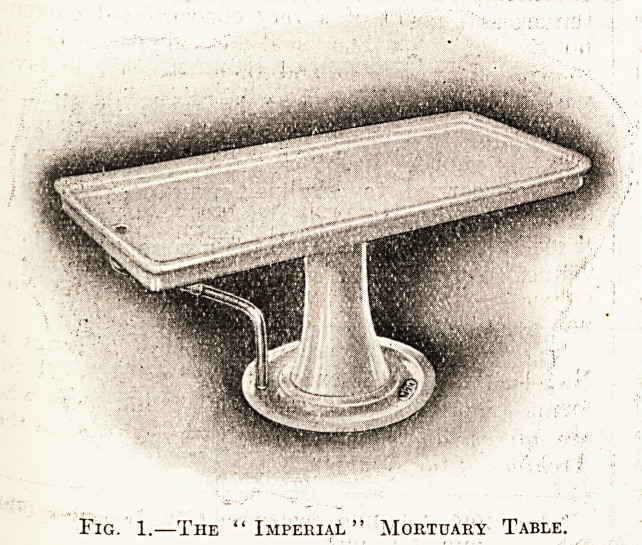


**Fig. 2. f2:**
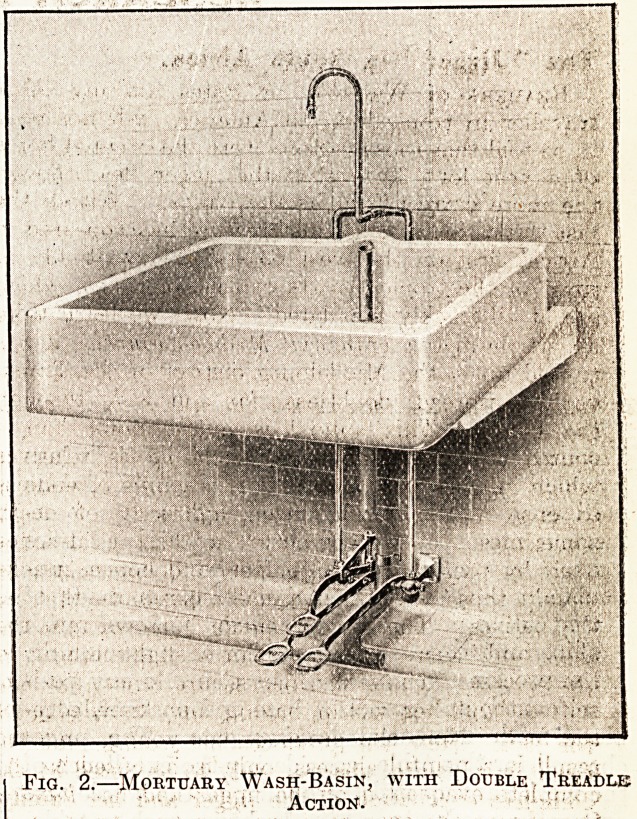


**Fig. 3. f3:**